# Plant cuticle research: reflections across two generations

**DOI:** 10.1093/jxb/erx393

**Published:** 2017-11-08

**Authors:** Martin J Bukovac, Antonio Heredia

**Affiliations:** 1 Department of Horticulture, Michigan State University, East Lansing, MI, USA; 2 IHSM-CSIC-UMA, Departamento de Bioquímica y Biología Molecular, Facultad de Ciencias, Universidad de Málaga, Campus de Teatinos, Málaga, Spain

Distinguished pioneer John Bukovac and Antonio Heredia reflect on more than half a century of cuticle research.

## In conversation with John Bukovac

An influential figure in plant cuticle research, John Bukovac began research in the 1950s following graduation from Michigan State University (MSU) (see Box 1 for further details).

**Box 1. John Bukovac and Antonio Heredia**

John and Antonio are pictured at the University of Málaga, Spain in 1994. An influential figure in plant cuticle research, John Bukovac began research in the 1950s following graduation from Michigan State University (MSU), where he majored in Horticulture. He had gone into military service, but Harold Tukey, chair of the department and a well known horticulturist/botanist, encouraged him to return and at the end of his service in 1953 he took up a ‘special research assistantship’ available in a grant from the Atomic Energy Commission. Even though Tukey was busy with administrative responsibilities, he served as his mentor, and Sylvan Wittwer, a relatively new faculty member who was actively researching foliar absorption of mineral nutrients, provided considerable additional guidance.

John worked at MSU for his entire career and was elected to the US National Academy of Sciences in 1983. During the 1970s and 80s, his lab became a focal point for young researchers, many from abroad, interested in the plant cuticle (Yasuyuki Yamada, Robert Norris, Jörg Schönherr, Edward Baker, Antonio Heredia, Peter Stevens and Moritz Knoche, to name a few). When they moved back to their home countries, they established their own research groups in different basic and applied research topics related to the cuticle, and many senior researchers from these countries could now also be considered disciples of his pioneering work. As a fitting flourish, in 1995 John received a senior Humboldt Award and went to Bonn (Germany) to work on fruit abscission in apple, going back to his horticultural beginnings. He was selected to receive an honorary doctorate. ‘Retiring’ in 1996, he returned to MSU and retained a small lab for several more years (as would so many research scientists given the chance), continuing a research programme on chemical control of flowering and fruit abscission in apple and cherry.

Antonio obtained his PhD at the University of Málaga in 1982 with a thesis in quantum chemistry. He began his research on the plant cuticle in 1988 at MSU with John Bukovac; his research over nearly three decades back at Málaga has kept that focus, revealing biophysical, structural and molecular aspects.

**Figure:**
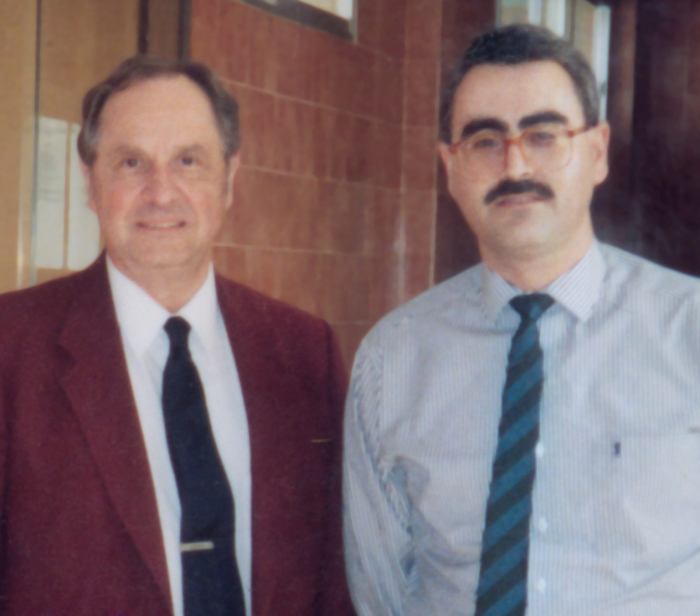

### What was your first area of research?

My research focused on absorption of atomic fission products by edible plants, mostly the common bean (*Phaseolus vulgaris*). The Atomic Energy Commission (AEC) provided me with soil samples collected on an atoll in the Pacific where atomic weapons were being tested: the contaminated samples were rich in Sr^90^ and Cs^137^. For root absorption, I labelled the soil media with a quantity of the radioactive soil. For foliar absorption, the AEC provided me with a solution of a soil extract, which I applied as various treatments to the surface of designated leaves. I followed penetration and translocation to the remainder of the plant, specifically the pods and seeds. These studies were not mechanistic.

### Can you talk about the key figures or labs in the field at that time?

When I started there were few labs in the US focusing on cuticular research, though several were working on foliar application of mineral nutrients, herbicides or plant growth regulators. The cuticle was quickly recognized as a barrier to penetration, but the excitement of being able to control weeds or induce numerous physiological processes in plants continued to dominate. I should mention that Harold Tukey [John’s mentor], together with Charles Hamner, published the first paper on selective action of 2,4-D in *Science* in 1944 (Hamner and Tukey, 1944).

Recognizing that the cuticle was a limiting factor, many researchers then focused on amending the aqueous spray solution with wetting agents/surfactants, ‘stickers’, slow-drying agents and other substances to increase penetration. Also during this period several large programmes emerged in the US, primarily on foliar uptake.

There are many individuals one could mention, and I was fortunate to work with a number of them. In 1965, I had decided to increase the basic aspect of my programme, and received a National Science Foundation Fellowship to work with Geoffery Blackman and John Martin, both in the UK. In Blackman’s lab at Oxford I established the effect of increasing chlorination (number and position) of phenoxyacetic and benzoic acids on penetration through enzymatically isolated tomato fruit cuticle – this was published in *Journal of Experimental Botany* (Bukovac *et al.*, 1971). While there, I also worked with Barrie Juniper and made some of the first carbon replicas of the inner and outer surfaces of isolated cuticular membranes. The carbon film was carefully removed in chloroform and then viewed in an electron microscope (a forerunner to scanning electron microscopy). With Martin and Edward Baker, at Long Ashton, I worked on isolation, fractionation and permeability of cuticular waxes. We plated artificial membranes with known quantities of each isolated wax fraction and then measured their permeability to 2,4-D in ‘home-made’ diffusion cells.

On my return to MSU, I focused on two areas: one on spray application and use of plant growth regulators to control flowering and fruiting in tree fruits (primarily cherry and apple), and a second on the structure, permeability and factors that affect sorption and penetration of the cuticle. This latter programme was designed to improve the horticultural use and performance of plant growth regulators.

### What about individuals in your own lab?

I was very fortunate in having many dedicated and capable students and postdocs. Too many to cover individually here. But it would be nice to mention my first postdoc, Yasuyuki Yamada. Early in my career (1958) I was invited to lecture at a UNESCO course held at Tokyo University. The primary purpose was to train scientists from southeast Asia in the use of radioisotopes in plant research. At the end of the course my host arranged for me to visit with a student who I had corresponded with (Yasuyuki) earlier. He was working on urea penetration. After graduation (1962) he joined our lab for three years and worked on penetration, binding and effects of urea on ion penetration. He was a very talented and pleasant colleague. On return he was the first to produce a rice plant from a protoplast. He also became the president of the Nara Institute of Science and Technology (NAIST) and the youngest member of the Japanese Academy of Sciences.

Another one to mention is Jörg Schönherr, who worked on factors that control stomatal entry, aqueous pores, ion exchange properties and diffusion kinetics. On his return to Germany he devoted his entire career to studies on the cuticle and, with a student, Lukas Schreiber, wrote a book on the permeability of plant cuticles (Schreiber and Schönherr, 2009). In the preface he writes that ‘The idea of analyzing permeability of cuticles based on structure–property relationships was born during a stay (1967–1972) by one of us (JS) as a doctoral student in Bukovac’s laboratory at Michigan State University, USA’. Many of my students and postdocs continued to do research on the cuticle and related problems after leaving my lab.

### Were there particular technological changes driving research?

The discovery of plant growth regulators/herbicides like 2,4-D, and related chemicals, initiated a keen interest in foliar penetration studies. The ability to visually observe and quantify a response following foliar application resulted in numerous publications documenting factors affecting foliar absorption, namely spray volume, concentration, surfactants and leaf characteristics (e.g. waxy vs pubescent). It was possible to obtain such data with limited technology and the results led to a rapid acceptance of foliar application, particularly in use of herbicides in agricultural practice.

With advances in technology after the late 1960s, research focused on the cuticle as a barrier to penetration, and our understanding of the cuticular membrane increased remarkably. With radioisotopes, it was possible to make quantitative measurements of sorption, desorption and penetration, and imaging of absorption and transport with autoradiography. With GC-MS (gas chromatograph coupled to mass spectrometer) chemical constituents of the cuticle could be identified. And with developments in microscopy there were many new findings: with the electron microscope, detailed transverse structure; with the scanning electron microscope, transverse and surface fine structure of the cuticle, epicuticular wax structure and density, deposition and surface distribution of many plant growth regulator deposits (and with a backscatter option, distribution of deposits containing heavy metals, and even crystallographic structure, if present); and with a stereo-scanning microscope, structure of the stomatal pore and the depth to which water of varying surface tension would penetrate.

### Can you describe some of the key questions at this point? Also which research changed our understanding?

Probably the most pressing questions were how to increase penetration of 2,4-D and related bioactive compounds, how the cuticle limits penetration, and how this can be overcome. Many researchers contributed to the general understanding by a variety of studies including spray retention, dosage, use of surfactants, surface wax abrasion and temperature. These were relatively simple studies, often using visual rating of plant injury or other visible symptoms.

Another question was whether stomata serve as a pathway for penetration. Schönherr’s work defining the role of surface tension and the architecture of the stomatal pore provided convincing information on how a liquid may enter via stomata (Schönherr and Bukovac, 1972). Interest in the cuticle *per se* soon led to studies on cuticle development, composition, embedded waxes and the cuticle cell/wall interface.

Last, are their cuticular pores that serve as pathways for penetration of polar and non-polar compounds? Studies by Schreiber (2005) and several other students of Schönherr provided convincing data for aqueous pores that accommodate hydrophilic molecules and the lipophilic cutin matrix that accommodates non-polar molecules.

### What would you consider the most important breakthroughs?

First, the discovery of 2,4-D and other bioactive compounds, primarily plant growth regulators (2,4-D being one). This had dramatic potential for agriculture. The bio-response induced could be quickly seen, and observed visually (recorded or photographed) or measured using simple devices. Individuals working with foliar nutrition did not have this advantage.

Other breakthroughs allowed research to flourish. Isolation of the non-living cuticle from many leaves and fruit of economic plants without (significant) loss of properties was very important. Further, the isolated cuticle could be stored and used later for analysis or permeability studies. Thus, the initial isolation of the cuticle with pectinase was most significant (Orgell, 1955).

And returning to technology, it is important to re-emphasize the importance of radioisotope technology. C^14^ labelling of many herbicides, plant growth regulators and mineral nutrients, and automated counting systems, made for quantitative analysis of sorption, desorption and penetration under many conditions. Advances in instrumentation continued to have an impact during my career, just as they do today.

### You retired in 1996. How would you reflect on progress at that time?

By the end of the 20th century, researchers had made significant advances in our knowledge of cuticle structure, composition and permeability, and on factors that affected sorption/desorption and trans-cuticular penetration, such as pH, concentration, molecular structure of the penetrant, and role of spray additives (e.g. surfactants, ammonium nitrate and spray retention agents).

During this period the agricultural chemical industry grew tremendously in the US, UK, France, Germany, Switzerland and Japan in parallel with cuticle research. Companies capitalized on use of the plant growth regulators which had been discovered and introduced numerous additional bioactive products. They further extended and optimized the findings of the researchers and developed useful and effective formulations of compounds for specific agricultural applications. Last, they modified the genetics of some plants, including corn and soybean, to permit a specific herbicide to kill weeds without affecting the crop itself.

## In conversation with Antonio Heredia

Antonio began his research on the plant cuticle in 1988 at MSU with John Bukovac (Box 1).

### Can you tell us about your link with John?

John Bukovac was an authority in the plant cuticle field. Not only because of his experience, but also the way he approached his subject from different angles and his ability to combine different perspectives. His kind and warm character and his attitude towards colleagues, students and staff have always been inspiring to me. Before his retirement, we applied for a NATO Collaborative Research Grant (1990–1994) that allowed a fruitful continuation of the research I started in his lab.

### And how would you reflect on his research today?

The areas that he has mentioned are still open – his research is very relevant in the field of applied agricultural science. There are a good number of researchers in agricultural companies and academia who worked and collaborated with John and who are currently involved or working on these topics. His lab was characterized by a combination of basic and highly applied research, and this imparted a broad perspective to the scientists ‘formed’ there.

### Considering your own experience, would you comment on the changing nature of research?

Research has certainly changed in the last two decades. The current acceleration of society is also affecting the way we do science and there is a lot of pressure, especially for young students and researchers. At the same time the techniques and methodologies for doing science have evolved tremendously, allowing us to do things we could only dream of just a few years ago. Essential questions about our research subject, the plant cuticle, still remain but the way they are being addressed has changed considerably. We have more precise and sophisticated equipment but we must continue to pursue the appropriate question for each approach to improve and increase our knowledge. There is so much information at our fingertips these days that the big challenge is to integrate all the information coming from different disciplines in order to improve our understanding of a research topic.

### Perhaps you could finish by bringing us briefly up to date on plant cuticle research?

More recent research, from this new century on, has been very fruitful. Some colleagues have focused their attention on biophysical properties. There has been noticeable progress in understanding the hydrodynamics, mechanics and thermodynamics of the cuticle, together with physiological implications. At the same time a lot of progress has been made in elucidating the complex architecture and structure of plant cuticle, an extraordinary, complex biopolymer. Moreover, during the past two decades there have been tremendous advances in understanding its molecular biology. Advances in the field of genetics and the development of collections of mutants have allowed the identification of many genes involved in the synthesis and regulation of cuticle components and more recently in cutin assembly. In this context, it is important to remember the pioneering work carried out in the 1970s and 80s by Pappachan Kolattukudy on the biochemistry of plant cuticle components, with special emphasis on the proteins involved in the synthesis of waxes, cutin monomers and the cutin polymer. Continuing with the more recent past, the switch in focus from Arabidopsis (almost exclusively) to the inclusion of several crops has added a plant breeding dimension to cuticle studies. Nevertheless, the research carried out with Arabidopsis is of great importance since it has given new perspectives to our understanding of the cuticle.

## References

[CIT0001] Bukovac MJ, Sargent JA, Powell RG, Blackman GE 1971 Studies on foliar penetration: VIII. Effects of chlorination on the movement of phenoxyacetic and benzoic acids through cuticles isolated from the fruits of *Lycopersicon esculentum* L. Journal of Experimental Botany 22, 598–612.

[CIT0002] Hamner CL, Tukey HB 1944 The herbicidal action of 2,4 dichlorophenoxyacetic and 2,4,5 trichlorophenoxyacetic acid on bindweed. Science 100, 154–155. 17778584 10.1126/science.100.2590.154

[CIT0003] Orgell WH 1955 The isolation of plant cuticle with pectic enzymes. Plant Physiology 30, 78–80. 16654733 10.1104/pp.30.1.78 PMC540601

[CIT0004] Schönherr J, Bukovac MJ 1972 Penetration of stomata by liquids: dependence on surface tension, wettability, and stomatal morphology. Plant Physiology 49, 813–819. 16658054 10.1104/pp.49.5.813 PMC366058

[CIT0005] Schreiber L 2005 Polar paths of diffusion across plant cuticles: new evidence for an old hypothesis. Annals of Botany 95, 1069–1073. 15797897 10.1093/aob/mci122 PMC4246894

[CIT0006] Schreiber L, Schönherr J 2009 Water and solute permeability of plant cuticles: measurement and data analysis. Springer.

